# Identifying Tumor-Associated Genes from Bilayer Networks of DNA Methylation Sites and RNAs

**DOI:** 10.3390/life13010076

**Published:** 2022-12-27

**Authors:** Xin-Jian Xu, Hong-Xiang Gao, Liu-Cun Zhu, Rui Zhu

**Affiliations:** 1 Department of Mathematics, Shanghai University, Shanghai 200444, China; 2 School of Life Sciences, Shanghai University, Shanghai 200444, China; 3 Department of Clinical Laboratory Medicine, Shanghai Tenth People’s Hospital of Tongji University, Shanghai 200444, China; 4 Department of Medical Biophysics, University of Toronto, Toronto, ON M5S 2E8, Canada

**Keywords:** bilayer network, centrality, DNA methylation, RNA

## Abstract

Network theory has attracted much attention from the biological community because of its high efficacy in identifying tumor-associated genes. However, most researchers have focused on single networks of single omics, which have less predictive power. With the available multiomics data, multilayer networks can now be used in molecular research. In this study, we achieved this with the construction of a bilayer network of DNA methylation sites and RNAs. We applied the network model to five types of tumor data to identify key genes associated with tumors. Compared with the single network, the proposed bilayer network resulted in more tumor-associated DNA methylation sites and genes, which we verified with prognostic and KEGG enrichment analyses.

## 1. Introduction

Biological processes include complex molecular interactions that can be efficiently characterized by network models [1]. Since the early 2000s, much attention has been paid to the structure and function of molecular interaction networks. Metabolic, protein, and gene coexpression networks possess small-world and scale-free characteristics [2,3,4,5,6]. Additionally, the robustness of the molecular interaction networks can help us understand the mechanisms behind complex diseases [7,8]. In particular, disease-associated genes need to be identified from the network perspective [9,10,11].

Although experimental methods can more accurately identify disease-associated genes, they are costly and time consuming [12]. Therefore, many computational approaches have been proposed to identify disease-associated genes, for example, through gene expression analysis or some machine learning methods [13,14]. However, these gene expression analyses are subjected to many uncertainties, and therefore they are not very accurate [15], but machine learning methods often lead to overfitting and the results cannot be adequately explained [16]. Because of their accuracy and interpretability, molecular interaction networks have attracted considerable attention from the biological community. Specifically constructing a proper network model and using it to identify disease-associated genes are fundamental problems. In the literature, many approaches have been developed through nodal centrality. For instance, the tumor-associated genes in gene coexpression networks have more edges than nonassociated genes, requiring the adoption of the degree centrality to identify these genes [17]. Additionally, the betweenness centrality, defined on the basis of shortest paths, has been adopted in protein–protein interaction networks to identify tumor-associated proteins [18]. On the basis of these two measures, many variants have been developed, such as the PageRank centrality [19] and the HITS algorithm [20]. The different centrality indicators enable the identification of hub nodes in the network from different perspectives [21].

In recent decades, multilayer networks have been widely used to thoroughly analyze biological systems because of the multiple and complex interactions between molecules [22,23]. The multilayer networks generated from multiomics provide more information than single networks [24]. For instance, Cantini et al. [25] constructed four-layer network including transcription factor cotargeting, microRNA cotargeting, protein–protein interactions, and gene coexpression, and showed that the multilayer network communities were enriched in biological components involved in the oncogenic process that could not be determined from the coexpression network alone. Ehsan et al. [26] applied the random walk algorithm to a three-layer network containing gene coexpression, regulatory, and physical interaction layers, and identified several hub genes affecting colon carcinoma. Mahapatra et al. [27] proposed a multiplex network model using gene expression and gene methylation data from oral cancer to identify hub nodes. During the analysis, the centrality measure was still used. Zhang et al. [28] built a miRNA–protein expression network for breast cancer patients and combined degree and betweenness centralities to find new miRNAs as biomarkers. Wang et al. [29] proposed EDCPID centrality based on tensor decomposition and applied it to a yeast landscape and H3N2 inflammatory and lung cancer multilayer networks, being able to effectively identify hubs. Chen et al. [30] calculated the betweenness centrality of the protein layer nodes in the network and filtered out the top 10% proteins in a four-layer network of ingredient–protein–metabolic pathway disease associated with the Xiaochaihu decoction.

Despite many studies on multilayer molecular interaction networks, we notice two shortcomings. First, in most multilayer network models (e.g., [26,28]), the focus has been gene and protein coupling, but few researchers have considered epigenomic data. Second, the construction of each layer of a multilayer network uses only the corresponding single omics, whereas the impact of other omics has usually been ignored (e.g., [25,26]). In this study, we used DNA methylation and RNA interaction data to construct a bilayer network of DNA methylation sites–RNAs. In particular, we considered the interactions between the genes corresponding to two DNA methylation sites in the RNA layer to determine whether the correlation between these two DNA methylation sites is reliable. Applying the method to four typical tumors, we identified more tumor-associated genes and DNA methylation sites in the network with centrality indicators. The results of the prognostic analysis of these hub nodes showed that disease-associated DNA methylation sites could be more accurately found using a bilayer network than with a single network. The results of KEGG pathway analysis confirmed that the hub genes identified through the bilayer network were closely associated with tumors.

## 2. Materials and Methods

### 2.1. Data Collection and Preprocessing

We obtained DNA methylation datasets (Illumina Human Methylation 450K, level 3), gene expression datasets (IlluminaHiSeq_RNASeqV2, level 3), and clinical datasets for lung squamous cell carcinoma (LUSC), breast cancer (BRCA), endometrioid cancer (UCEC), kidney cancer (KIRC), and bladder cancer (BLCA) from the TCGA database [31]. The β values were derived at the Johns Hopkins University and University of Southern California TCGA genome characterization center, which are continuous variables between 0 and 1, representing the ratio of the intensity of the methylated bead type to the combined locus intensity [32]; we removed the probes with β values of “NA” and those not in the gene regions considered in our analysis. Clinical data included time of death and death status of patients (Table S1).

We obtained the RNA binding relationships from the RNAInter database [33], which is a comprehensive RNA interactome resource. The database scores the confidence level of each interaction relationship by combining literature support and experimental validation, where a score above 0.75 indicates the existence of strong experimental evidence for the interaction relationship [34].

### 2.2. Network Construction

Currently, DNA methylation networks are generally constructed on the basis of the correlation coefficients between sites [35]. However, DNA methylation sites mainly regulate gene expression by recruiting proteins involved in gene expression or by inhibiting the binding of transcription factors to DNA, with no direct relationship between the sites [36]. Therefore, networks based on Pearson correlation coefficients between the sites are inaccurate. The methylation level of a site affects the expression of the corresponding genes [37], which motivated us to construct a bilayer network of DNA methylation sites and RNAs.

#### 2.2.1. Construction of the DNA Methylation and RNA Interaction Networks

We determined the differences in the DNA methylation sites between tumor and paraneoplastic tissue using empirical Bayes’ moderated t-test method, contained in the limma package [38] in R (version 4.0.3, Guido Masarotto, AT, 2020). To reduce the risk of false positives, we adjusted p-values with the Benjamini–Hochberg false discovery rate (FDR) method. We used an FDR < 0.01 as the significance threshold [39]. We then joined the edges using the cutoff of the Pearson’s correlation coefficients between sites. The Pearson correlation coefficient [40] between the different DNA methylated sites was defined as follows:

(1) $$r=\frac{\sum_{i=1}^{n}\left(X_{i}-\bar{X}\right)\left(Y_{i}-\bar{Y}\right)}{\sqrt{\sum_{i=1}^{n}{\left(X_{i}-\bar{X}\right)}^{2}}\sqrt{\sum_{i=1}^{n}{\left(Y_{i}-\bar{Y}\right)}^{2}}}$$

where n is the number of samples in the tumor dataset, $X_{i}$ is the level of the DNA methylation of the $i_{th}$ sample, and $\bar{X}$ is the average level of the DNA methylation at the site. We calculated all r values with the C language program. The screening of edges in correlation networks is mainly based on the hypothesis test p-value or the cutoff of correlation coefficients. Because the p-value is easily affected by the sample size [41], we adopted a cutoff of the correlation coefficients to construct the DNA methylation network. Here, two DNA methylation sites were linked if the correlation coefficient |r|>0.8 [42].

RNAs can regulate each other through binding relationships [43], which mainly depend on the structure of the RNA and the base sequence [44] and are relatively stable [45]. For the RNA layer, we therefore used the binding relationship between RNAs. For the edges of the RNA layer, we used the RNA interactions with confidence scores >0.75 from the RNAInter database, which are supported by strong experimental evidence [34].

#### 2.2.2. Construction of Bilayer Network of DNA Methylation Sites–RNAs

The links between the DNA methylation layer and the RNA layer are the DNA methylation sites connected with the corresponding genes. Using the edge information in the RNA layer, we filtered the edges in the DNA methylation layer. Specifically, we required two methylation sites to satisfy one of the following conditions: (i) being located on the same gene; (ii) the corresponding genes connected at the RNA layer; and (iii) the corresponding genes did not have an edge in the RNA layer, but they connected to an intermediate gene. Edges between DNA methylation sites that did not meet one of the above conditions were removed, even though they were strongly correlated with each other. The above criteria were determined using the networkX package in python (version 3.8.1).

### 2.3. Network Indicators

#### 2.3.1. Degree Centrality (DC)

**Figure 1:**
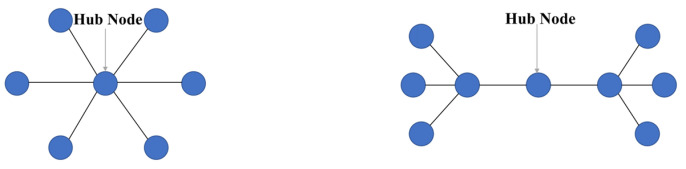
Illustration of a hub node based on the (**left**) degree and (**right**) betweenness centralities.

Degree centrality [46] represents the number of connected edges that a node has with other nodes in the network. The degree centrality of node $v_{i}$ is defined as

(2) $$DC\left(v_{i}\right)=\frac{\sum_{j}A_{ij}}{N-1}$$

where N is the total number of nodes in the network, and A  represents the adjacency matrix of the network. The larger the degree of centrality of a node, the more important it is the in the network [3] (Figure 1).

#### 2.3.2. Betweenness Centrality (BC)

Betweenness centrality [47] is a measure of the participation of a node in the shortest paths in a network. The betweenness centrality of node $v_{i}$ is defined as

(3) $$BC\left(v_{i}\right)=\underset{V_{S}\neq V_{i}\neq V_{t},s<t}{\sum }\frac{\sigma_{st}\left(v_{i}\right)}{\sigma_{st}}$$

where $\sigma_{st}$ is the number of shortest paths from s to t, and $\sigma_{st}\left(v_{i}\right)$ is the number of shortest paths from s to t that pass through node $v_{i}$. In biological networks, nodes with high betweenness centrality generally play a key role in the connectivity of the network, such as communicating two modules and serving as a bridge to connect them [48] (Figure 1).

In this study, only the DNA methylation layer was different in the bilayer network for various tumors, and therefore we performed the centrality analysis only for the DNA methylation layer.

#### 2.3.3. Average Degree

The average degree, usually denoted by 〈k〉, is the average of the degrees of all nodes [49]:

(4) $$\langle k\rangle =\frac{\sum A_{ij}}{N}$$

The average degree can indicate how many neighbors the nodes have, on average, in the network.

#### 2.3.4. ER Random Network

The ER random network model [49] is an equal-opportunity network model. In this model, given a certain number of nodes, a node has the same probability of inter-relationship (connection) with any other node, denoted as the edge probability p of the network.

#### 2.3.5. Clustering Coefficient

The clustering coefficient [49] is a coefficient used to describe the level to which nodes in a graph form clusters with each other. It considers not only the number of neighbors of a node, but also the relationships between neighboring nodes. For example, the number of neighbors of node i is $k_{i}$. These neighboring nodes have at most $k_{i}\left(k_{i}-1\right)/2$ edges between them. The clustering coefficient $C_{i}$ for node i is defined as the ratio of the number of edges $E_{i}$ formed between the neighboring nodes of that node and the maximum number of possible edges:

(5) $$C_{i}=\frac{2E_{i}}{k_{i}\left(k_{i}-1\right)}$$

The higher the clustering coefficient, the more compact the network. The average clustering coefficient $C_{network}$ is the average of the clustering coefficients of all nodes in the network. In biological networks, variations in the clustering coefficient are generally used to characterize the degree of modularity of the network [50].

#### 2.3.6. Shortest Path Length

The shortest path length, $d_{ij}$ between nodes i and j is defined as the number of edges on the shortest path connecting these two nodes [49]. The average shortest path is defined as the average of the paths between any two nodes in the network, and it reflects the tightness of the network:

(6) $$l_{network}=\frac{1}{\frac{1}{2}N\left(N+1\right)}\underset{i\geq j}{\sum }d_{ij}$$

The concept of the shortest path was used to find functional clusters in biological systems [51].

We calculated all the above metrics in a network using the networkX package in python (version 3.8.1).

### 2.4. Statistical Analysis

#### 2.4.1. Chi-Squared Test

The chi-squared test is used to test the level of deviation between the actual observed and theoretically inferred values of a sample [52]. The null hypothesis is that the observed frequencies do not differ from the expected frequencies, and the alternative hypothesis is that the observed frequencies differ from the expected frequencies. The chi-squared test statistic is defined as

(7) $$\chi^{2}=\sum \frac{{\left(f_{o}-f_{e}\right)}^{2}}{f_{e}}$$

where $f_{o}$ and $f_{e}$ represent the observed and theoretical values, respectively. For the chi-squared test of column independence, the degrees of freedom are $d_{f}=\left(R-1\right)\left(C-1\right)$, where R and C denote the number of rows and columns in the table, respectively. The chi-squared test requires the degree of freedom to determine the significance level of the statistic. The chi-squared table or statistical software can be used calculate the corresponding *p* value according to the chi-squared value and the degree of freedom. We conducted the chi-squared test in this study with the CHISQ.TEST function in Excel [53]. We considered *p*  <  0.05 as statistically significant.

#### 2.4.2. Log Rank Test

The log rank test is used to test the significant differences in the location of the distribution of the overall population in which the test data are located in the case of an arbitrary overall distribution [54]. By arranging the observations in ascending order, each observation is numbered in order, which is called the rank. The test is then performed by calculating the rank sum for each of the two groups of observations. The null hypothesis is that the overall distribution of the two groups is the same, and the alternative hypothesis is that the overall distribution of the two groups is different. The rank sum of the smallest group of sample size is used as the t-test statistic. In this study, we performed the log rank test using the lifelines package in Python (version 3.8.1). We considered *p*  <  0.05 as statistically significant, indicating the distribution of the two groups was significantly different.

### 2.5. Identification of Differentially Expressed Genes (DEGs)

We determined the differentially expressed genes between the tumor and paraneoplastic tissues using the empirical Bayes’ moderated t-test method, contained in the limma package [38] in R (version 4.0.3). We calculated log2 (fold change) using the average expression of the two groups of genes. The thresholds were FDR < 0.05 and | log2 (fold change) | > 1 [55].

### 2.6. Survival Analysis

We used survival analysis to examine the relationship between the DNA methylation sites and overall survival (OS). We divided patients into high- and low-risk groups on the basis of the mean β value of the site. We analyzed the difference between the two groups with KM analysis [56] on the basis of the lifelines package in Python (version 3.8.1). A log rank *p* < 0.05 was considered statistically significant.

### 2.7. KEGG Pathway Enrichment Analysis

We performed functional enrichment analysis for genes that we found only in the bilayer network. We used the Database for Annotation, Visualization, and Integrated Discovery (DAVID) [57] tool for the KEGG enrichment analysis based on the hypergeometric distribution to compute the p-values [58]. We set p<0.05 as the threshold value.

## 3. Results

### 3.1. Characteristics of DNA Methylation Sites–RNAs Bilayer Network

**Figure 2:**
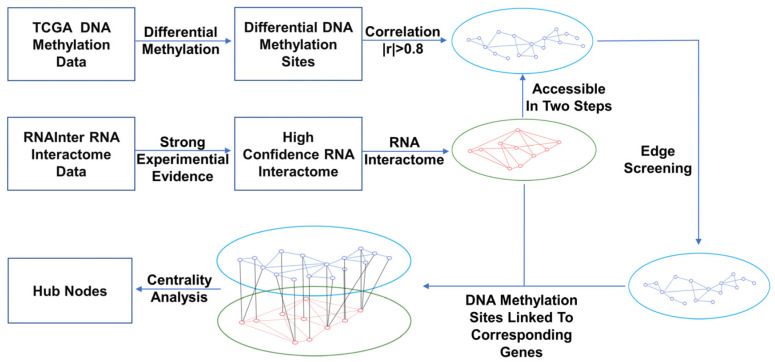
Flow of DNA methylation–RNA bilayer network analysis. Nodes in DNA methylation layer are blue, representing DNA methylation sites; nodes in the RNA layer are red, representing RNAs. We constructed the DNA methylation network on the basis of the cut-off correlation coefficient and the RNA network on the basis of RNA binding. According to the relationship between the genes corresponding to DNA methylation sites in the RNA layer, we performed edge filtering between the DNA methylation sites. After the bilayer network was constructed, we analyzed the hub nodes according to centrality indices.

On the basis of the DNA methylation data from the TCGA database and the RNA interaction information from the RNAInter database, we obtained bilayer networks of the DNA methylation sites and RNAs (Figure 2) for five types of tumors: LUSC, BRCA, UCEC, KIRC, and BLCA.

**Figure 3:**
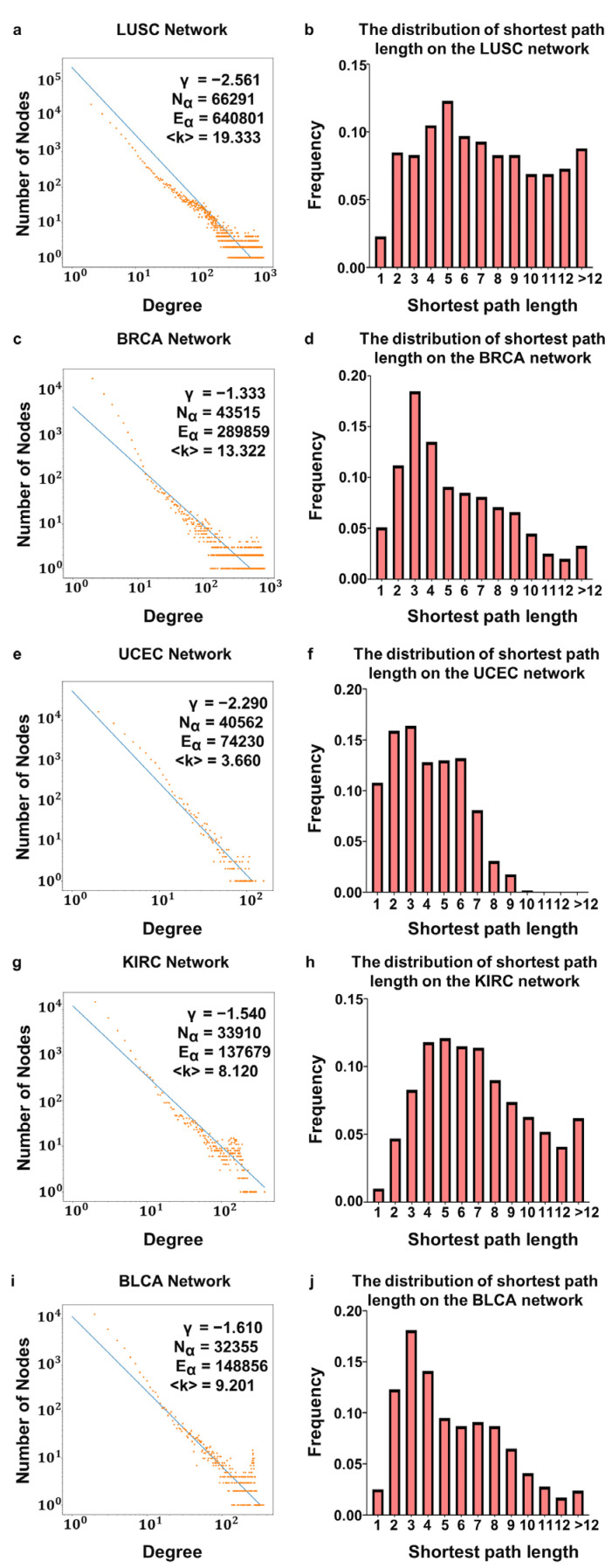
Distributions of nodal degree distributions and shortest path lengths for five datasets: (**a**,**f**) LUSC; (**b**,**g**) BRCA; (**c**,**h**) UCEC; (**d**,**i**) KIRC; and (**e**,**j**) BLCA. $N_{\alpha }$ represents number of nodes in the network, $E_{\alpha }$ represents number of edges in the network, and <k> represents average degree of nodes in the network.

Although the average degree in the DNA methylation layer considerably varied (e.g., 19 for LUSC compared with 4 for UCEC), the degree distributions of the five tumors showed right-skewed behavior, implying a scale-free characteristic (Figure 3). In this scenario, a few nodes in the network had a large number of edges, and thus they were identified as hubs. We also noticed the small-world property. As shown in Table 1, the average clustering coefficient was high, and the average shortest path length was low. We obtained all the results from the edge-filtered DNA methylation layer, which was much sparser than the single DNA methylation network directly generated from correlations.

Next, we analyzed the structural properties of the RNA layer. Despite various tumors, the structure remained unchanged, containing 8087 RNAs and 20,128 RNA binding relationships. Most of nodes in the network were mRNAs, and most of edges were produced between noncoding RNAs and mRNAs, in agreement with the situation in reality. Moreover, the average numbers of edges connected to lncRNA, miRNA, and mRNA were 15.207, 12.941, and 2.883, respectively, implying that the noncoding RNAs were more central. Finally, we recovered the scale-free and small-world features of the RNA network.

### 3.2. Correlation of Hubs with Tumor Development Process

Hub nodes play an important role in biological processes [58]. To identify these nodes, various centrality metrics have been adopted in the study of biological networks [3,59], among which the degree centrality [46] and betweenness centrality [47] are commonly used because of their efficacy and interpretability. Next, we applied these two centralities to rank the nodes with importance in the DNA methylation layer, and we present the most important nodes in Table 2.

According to screening based on the degree centrality, the most critical node for LUSC was Cg25080152, corresponding to the gene *MYC*, which is a target gene for cancer therapy [60]. The most important node we identified in BRCA was Cg24771570, which corresponds to the gene *GRB2*. Most cancer malignancies are caused by abnormal signaling of the Grb2 adaptor molecule [61]. Cg14751398 was the largest hub node in the UCEC network, located on *E2F3*, which is linked to poor prognosis in some cancers as an oncogenic factor [62]. Cg08311343 was the most significant node in the KIRC network, which is located on the *CDK6* gene. *CDK6* is able to regulate the cell cycle, and its inhibitors have been used as effective therapeutic drugs for breast cancer [63]. In the BLCA network, the most critical DNA methylation site was Cg12931157, corresponding to the gene *NFYA*, which is associated with cell-cycle alterations and cell proliferation as a transcription factor and is closely related to several tumors [64].

Our ranking of nodes with importance based on the betweenness centrality revealed that the most important node for LUSC was Cg08133058, corresponding to the gene *SASH1*, which is a prognostic indicator and a potential therapeutic target in non-small-cell lung cancer [65]. We identified Cg26383454, located on the *SMIM13* gene, as the most important DNA methylation site for BRCA, which is a membrane-associated protein. The key node identified for UCEC was Cg18776056, located on the gene *FKBP4*, which is a progestin receptor cochaperone protein associated with cancer malignancy [66]. We identified Cg19858017 for KIRC, corresponding to the gene *CLSTN1*, which can be used as a biomarker for a variety of cancers [67,68]. Finally, the most critical node in the BLCA network was Cg01473187, corresponding to the gene *TSPAN6*, which is a suppressor of Ras-driven cancer [69]. In summary, all the genes corresponding to the hub nodes identified were closely associated with tumors according to the two centrality measures.

**Figure 4:**
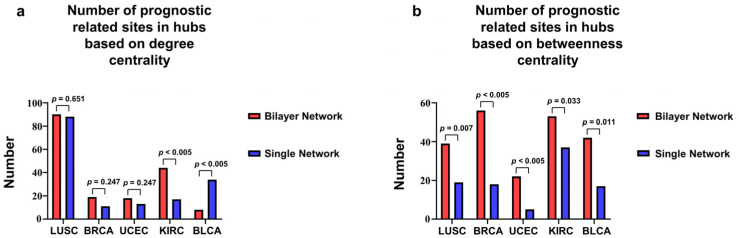
Number of survival-associated sites in the top 100 DNA methylation sites resulting from degree and betweenness centralities. (**a**) Comparison of the number of survival-associated sites in the top 100 DNA methylation sites according to degree centrality for bilayer and single methylation networks (chi-squared test LUSC $\chi_{p}^{2}=0.651$, chi-squared test BRCA $\chi_{p}^{2}=0.247$, chi-squared test UCEC $\chi_{p}^{2}=0.247$, chi-squared test KIRC $\chi_{p}^{2}<0.005$, and chi-squared test BLCA $\chi_{p}^{2}<0.005$). (**b**) Comparison of number of survival-associated sites in top 100 DNA methylation sites according to betweenness centrality for bilayer and single methylation networks (chi-squared test LUSC $\chi_{p}^{2}=0.007$, chi-squared test BRCA $\chi_{p}^{2}<0.005$, chi-squared test UCEC $\chi_{p}^{2}<0.005$, chi-squared test KIRC $\chi_{p}^{2}=0.033$, and chi-squared test BLCA $\chi_{p}^{2}=0.011$).

To illustrate the fact that the bilayer network could find more tumor-associated DNA methylation sites than the single DNA methylation correlation networks, we further compared the number of prognostically correlated loci among the hub DNA methylation sites found by the two approaches [34]. We calculated the number of survival-associated loci among the top 100–500 DNA methylation sites with the largest degree and betweenness centralities for the five tumor datasets (Figure 4, Table S2). In this scenario, the results of the chi-squared test for all the tumors showed that the betweenness centrality for the bilayer network was better than that of the single network because more prognostic-associated DNA methylation sites were identified. For the degree centrality, although the chi-squared test results showed that the bilayer network was better than the single network only for one cancer, the rest of the bilayer networks marginally outperformed the corresponding single network. This finding can be explained as a result of the filtering of edges between the DNA methylation sites through the information in the RNA layer, which enhanced the authenticity of the network. Thus, the betweenness centrality of the bilayer network was substantially improved compared with that of the single network. The degree centrality ranks the importance of a node by the number of its neighbors. In a biological network, the more a node interacts with other nodes, the more important the node. In contrast, the betweenness centrality assesses node importance by counting the number of times that it serves as the shortest path in the network. In a biological network, this shortest path is closely related to actual biological pathways. A node with a large betweenness centrality is located at the intersection of multiple critical pathways in the DNA methylation layer, and a contiguous edge in the methylation layer represents a pathway in the RNA layer where the node is also at the intersection of critical pathways, and therefore the identified node is more important to the network.

### 3.3. Correlations between DNA Methylation Sites Located on the Same Gene

**Figure 5:**
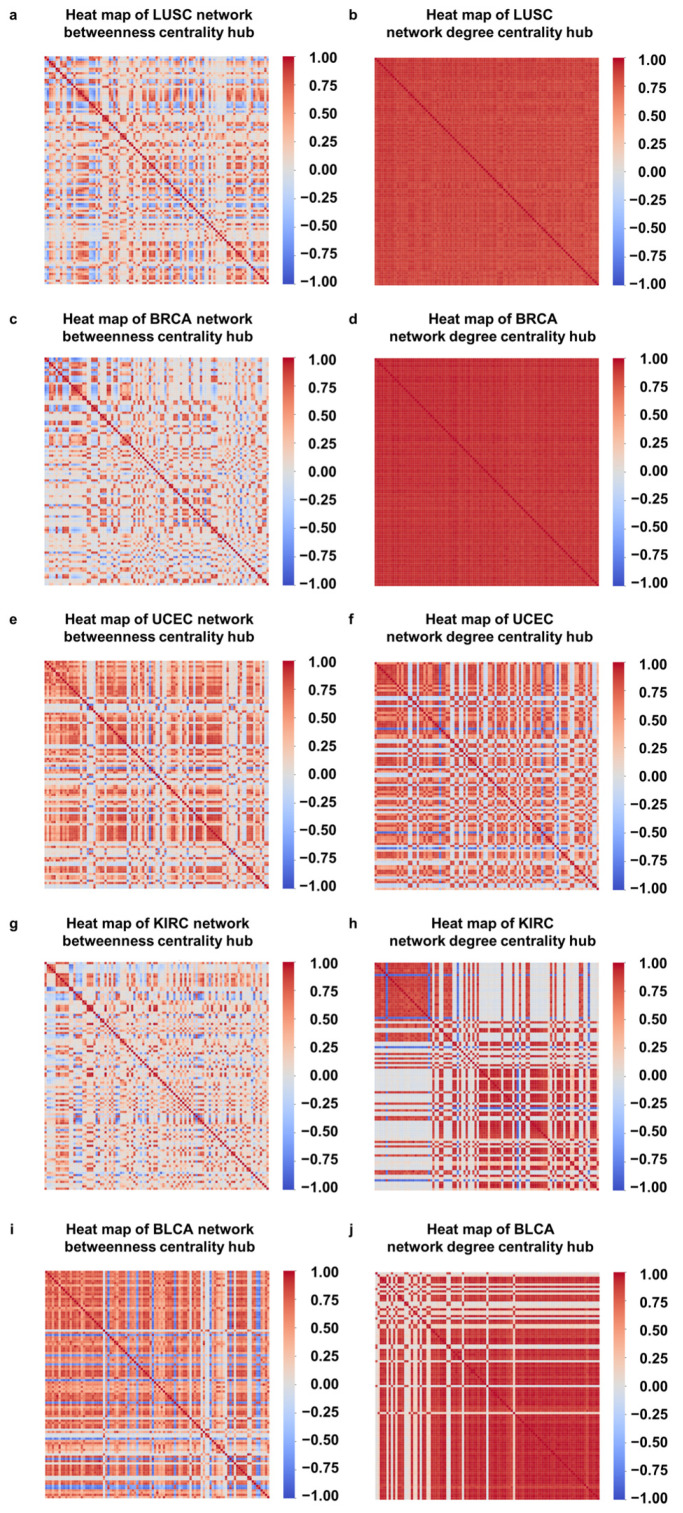
Correlation between top 100 DNA methylation sites in betweenness centrality and degree centrality rankings. Heat maps of degree centrality and betweenness centrality hubs of the same cancer are at the top and bottom of the same column: (**a**,**f**) LUSC; (**b**,**g**) BRCA; (**c**,**h**) UCEC; (**d**,**i**) KIRC; and (**e**,**j**) BLCA. The correlation between hub DNA methylation sites obtained by degree centrality was stronger, and the strong correlation between the hub DNA methylation sites obtained by betweenness centrality can also be observed.

In general, involvement in the same biological process or similarity in gene function leads to gene coexpression. To verify this property for comethylation [70], we calculated the correlation coefficients between hub the DNA methylation sites and present the heat maps in Figure 5 and the subnet formed by these hubs in Figures S1–S10. Overall, we noticed a strong correlation between them, implying that the identified hubs from the network corresponding to genes are likely located within the same biological pathways or perform similar functions. Using prognostic analysis and subsequent pathway enrichment analysis, we found that many of the hub nodes are associated with cancer.

For the DNA methylation sites on the same gene, the correlation between them tended to be stronger. For example, among the top 100 DNA methylation sites in the BRCA network, multiple sites, such as cg27588093, cg21160149, cg04988794, cg27523417, and cg17421241, are all located on the *PRDM16* gene, and we found a strong Pearson correlation coefficient between them, ${\left|\;r\;\right|}_{avg}=0.781$ (the average correlation of the top 100 DNA methylation sites resulting from the betweenness centrality was 0.277). This gene is a protein-coding gene that encodes a zinc finger transcription factor that suppresses tumor production [71]. Among the top 100 DNA methylation sites in the BLCA network, multiple sites, such as cg24701780, cg24804145, cg15192120, and cg25497530, are all located on the *PTPRN2* gene, and the average correlation between them was ${\left|\;r\;\right|}_{avg}=0.737$, which was larger than that averaged over the top 100 DNA methylation sites. This gene encodes a protein with sequence similarity to the receptor-like protein tyrosine phosphatase, which accelerates cancer progression and metastasis [72,73]. In Table S3, we summarize the correlation coefficients for the sites on the same gene. In general, they are stronger than those of the top 100 sites, in agreement with the literature [74].

### 3.4. Hubs in DNA Methylation Layer Aggregates Differentially Expressed Genes

To obtain deeper insight into the screened DNA methylation sites and their corresponding genes, we counted the number of differentially expressed genes near the top 100–500 DNA methylation sites ranked by degree centrality and betweenness centrality, separately. We found that the genes corresponding to the DNA methylation sites screened using either measure in the bilayer networks were accessible in two steps to the differentially expressed genes. On the contrary, only a small fraction of the genes corresponding to sites obtained from the single network could reach the differentially expressed genes in two steps. We show the results in Table 3 and Table S4. All tumor data differed, according to the chi-squared test, in both single and bilayer networks ($\chi_{p}^{2}<0.001$). The hub nodes in the DNA methylation layer of the bilayer network aggregated differentially expressed genes. As suggested by Le et al. [11], differentially expressed genes play a crucial role in tumor development.

However, this clustering of differentially expressed genes does not mean that all genes corresponding to hub DNA methylation sites are differentially expressed. The genes corresponding to the top 100 DNA methylation sites according to the centrality ranking are rarely differentially expressed and most of them are linked to differentially expressed genes through some noncoding RNA. For example, in the top 100 sites for LUSC, most genes are related to SNHG16 or some other miRNA. SNHG16 is a lncRNA regulating a number of mRNAs in the RNA layer that can be reached in two steps via SNHG16. Because noncoding RNAs regulate multiple mRNAs and mRNAs are regulated by multiple noncoding RNAs, these noncoding RNAs act as bridges between the mRNAs in the RNA layer.

The larger the betweenness centrality of a node, the higher the number of shortest paths traveling through that node. Therefore, the betweenness centrality can be used to identify hub nodes that are located in key pathways in the network that are likely to be involved in the expression of differential genes. For the degree centrality, the hubs identified on the basis of it are more likely to interact with differential genes because they have many neighbors. Similar to the results of the prognostic analysis, the results of the differential expression analysis were also better under the betweenness centrality than under the degree centrality. All genes corresponding to the top 500 DNA methylation sites according to the betweenness centrality reached the differentially expressed genes in two steps for all tumors. Under the degree centrality, only four tumors exhibited the same behavior.

### 3.5. KEGG Pathway Analysis

**Figure 6:**
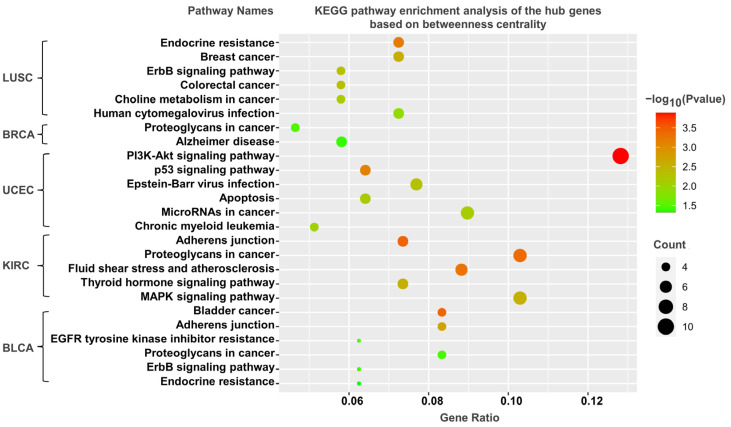
Gene-enriched pathways screened from bilayer networks according to betweenness centrality. The X-axis represents the count and ratio of hub genes, dot size represents the number of hub genes, and dot color represents the negative of the logarithm of the *p*-value.

**Figure 7:**
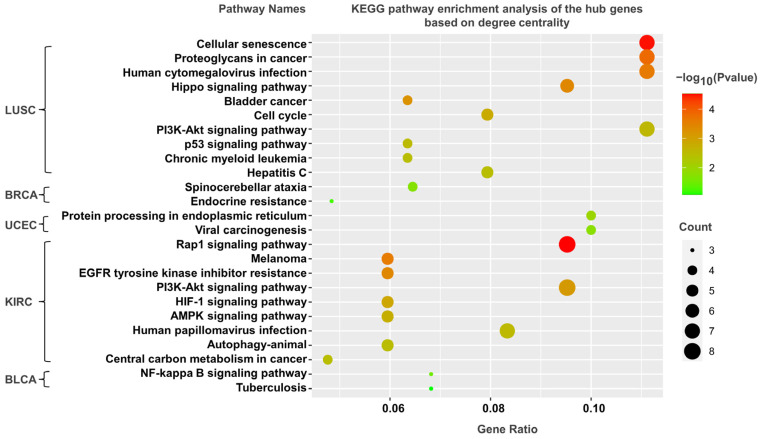
Gene-enriched KEGG pathways screened from bilayer networks according to degree centrality. The X-axis represents count and ratio of hub genes, dot size represents the number of hub genes, and dot color represents the negative of the logarithm of the *p*-value.

As mentioned above, the genes corresponding to the top 100–500 DNA methylation sites in terms of the degree and betweenness centralities could be screened in both the bilayer and single networks. The shortest paths in the network are closely related to biological pathways. The genes identified by the betweenness centrality have a high probability of being located in the critical pathway. Analogously, the genes identified on the basis of the degree centrality are likely to be located on some critical pathways due to their large number of neighbors. To explore the functions of those genes, we performed KEGG pathway enrichment analysis (Figure 6 and Figure 7, Table S5).

Figure 6 shows the KEGG pathway enrichment results of screening the top 100 genes on the basis of the betweenness centrality, where multiple KEGG pathways are associated with tumors. For hub genes in the bladder cancer bilayer network, the most important pathway is “bladder cancer”, in addition to “adherens junction” and “proteoglycans in cancer”. The hub genes in the endometrioid cancer bilayer network are enriched in the “PI3K–Akt signaling pathway” and “p53 signaling pathway”. The p53 signaling pathway is one of the most well-known cancer-related pathways, playing an integral role in multiple tumors [75]. Proteoglycans in cancer is an important cancer-related pathway closely related to the immune escape of tumor cells [76]. The PI3K-Akt signaling pathway plays an essential role in the regulation of cell survival, growth, and proliferation [77]. Moreover, several cancer-related pathways are also enriched, including “pathways in cancer”, “bladder cancer”, and “microRNAs in cancer”.

The enrichment results of the top 100 screened genes based on the degree centrality are shown in Figure 7. We likewise identified cancer-related pathways, such as “hippo signaling pathway”, “central carbon metabolism in cancer”, “PI3K-Akt signaling pathway”, and “bladder cancer”. In summary, the bilayer network approach could find genes involved in tumor-related processes that could not be found by the single networks.

## 4. Discussion

DNA methylation can influence life processes by regulating gene expression and is therefore associated with the development of various tumors [70]. In this study, we constructed a bilayer network using DNA methylation and RNA interaction data from five tumors and identified a set of hub DNA methylation sites and genes using centrality indicators. Both the DNA methylation and the RNA layer networks showed the scale-free and small-world characteristics that are essential in biological networks. The majority of the DNA methylation sites screened using the centrality metric were also associated with prognosis, and the bilayer network outperformed the single network, enabling the identification of more prognosis-associated sites. By analyzing the correlation between the DNA methylation sites, we illustrated that the sites on the same gene are more strongly correlated. In addition, we found that differentially expressed genes near the hub sites were enriched. Finally, our KEGG analysis revealed that hub genes in the RNA layer were involved in multiple tumor-related pathways.

Regarding the hub nodes identified in the DNA methylation layer, several issues are worth discussing. First, the genes corresponding to the most critical DNA methylation sites were mRNAs, which are closely associated with tumors. For the RNA layer, however, noncoding RNAs are located at more central positions. This is partly due to most of the DNA methylation sites being located on protein-coding genes. We found that 298,715 DNA methylation sites are in the gene region, of which 297,057 are on mRNA and only 1658 are on noncoding RNAs such as lncRNA. Additionally, in the RNA layer, a noncoding RNA can act as a bridge between two mRNAs, which results in the two mRNAs being accessible in two steps, and the DNA methylation sites on these mRNAs are not filtered out. Second, because the DNA methylation sites on the genes that perform similar functions are comethylated, we calculated the correlations between the hub sites, finding that these hubs always showed more positive correlations between them. Although DNA methylation sites do not directly interact, the positive correlation between the hub sites is actually a reflection of the site–gene–gene–site relationship. We speculate that the reason for this positive correlation may be related to the regulation of gene expression by DNA methylation sites as well as post-transcriptional regulation. A combination of site–gene correlations as well as gene–gene correlations may be required to explain this finding.

In the RNA layer, we found that noncoding RNAs act as key bridges in the network. These noncoding RNAs contain lncRNAs and miRNAs, with a smaller number of noncoding RNAs at the central of the network and a larger number of mRNAs at the margin of the network. Only 7 pairs of mRNAs are directly linked, whereas 5,981,746 pairs of mRNAs are indirectly linked through a noncoding RNA. Therefore, the correction of the RNA layer to the DNA methylation layer is affected only by noncoding RNA. The difference in the status of noncoding RNAs and mRNAs also shows that our network is able to allow for some errors in the RNA layer.

Overall, the proposed bilayer network framework has higher fidelity than traditional correlation networks and can be used to effectively analyze multi-mics data to identify many tumor-associated DNA methylation sites and genes that cannot be identified by single networks. We suggest three avenues for future study. First, the methylation of loci is mainly regulated by methylation-modifying proteins, which we did not consider in this study. Protein layers can be incorporated into our multilayer network. Second, we used only TCGA data in the present study, and other data sources may be added for validation in a subsequent study. Finally, for the identification of hub nodes in the network, we only used two typical centrality metrics. Other popular metrics are worth considering.

## Figures and Tables

**Table 1:** Information of the DNA methylation layer network.

Tumor Type	Number of Nodes in the DNA Methylation Layer	Number of Edges in the DNA Methylation Layer	Average Degree	Average Clustering	Average Clustering of the ER Network	Average Path Length	Average Path Length of the ER Network
LUSC	66,291	640,801	19.333	0.451	0.003	8.131	11.102
BRCA	43,515	289,859	13.322	0.436	0.003	6.227	10.681
UCEC	40,562	74,230	3.660	0.495	0.001	4.559	10.611
KIRC	33,910	137,679	8.120	0.430	0.002	6.907	10.431
BLCA	32,355	148,856	9.201	0.466	0.002	5.596	10.385

**Table 2:** Hub nodes and corresponding genes screened according to centrality metrics.

	Degree		Betweenness	
Tumor Type	Hub DNA Methylation Sites	Corresponding Gene	Hub DNA Methylation Sites	Corresponding Gene
LUSC	Cg25080152	*MYC*	Cg08133058	*SASH1*
BRCA	Cg24771570	*GRB2*	Cg26383454	*SMIM13*
UCEC	Cg14751398	*E2F3*	Cg18776056	*FKBP4*
KIRC	Cg08311343	*CDK6*	Cg19858017	*CLSTN1*
BLCA	Cg12931157	*NFYA*	Cg01473187	*TSPAN6*

**Table 3:** Number of DNA methylation sites near differentially expressed genes.

	Degree		Betweenness	
Tumor Type	Single Network	Bilayer Network	Single Network	Bilayer Network
LUSC	55	100	60	100
BRCA	51	100	51	100
UCEC	70	100	63	100
KIRC	63	100	59	100
BLCA	54	100	54	100

## Data Availability

We used public data accessible from the Cancer Genome Atlas (TCGA) database and RNAInter database.

## Supplementary Materials

The following supporting information can be downloaded at https://www.mdpi.com/article/10.3390/life13010076/s1. Table S1: Basic TCGA data information. Table S2: Comparison of prognostic correlation numbers of DNA methylation sites in top 100 to top 500 centrality. Table S3: Correlation between DNA methylation sites on the same gene. Table S4: Number of differential genes within two steps of DNA methylation sites of top 100 to top 500 centrality. Table S5: KEGG enrichment results of hub genes in top 100 to top 500. Figures S1–S10: Hub nodes found by degree and betweenness centralities constituting the subnetwork of the five tumors.

## References

[1] Barabási A.L., Gulbahce N., Loscalzo J. (2011). Network medicine: A network-based approach to human disease. Nat. Rev. Genet..

[2] Smith H.B., Kim H., Walker S.I. (2021). Scarcity of scale-free topology is universal across biochemical networks. Sci. Rep..

[3] Jeong H., Mason S.P., Barabási A.L., Oltvai Z.N. (2001). Lethality and centrality in protein networks. Nature.

[4] Naderi Y.P., Richardson C., Saule E., Loraine A., Taghi M.M. (2020). Revisiting the use of graph centrality models in biological pathway analysis. BioData Min..

[5] Das A.B. (2020). Small-world networks of prognostic genes associated with lung adenocarcinoma development. Genomics.

[6] Van D.S., Võsa U., Vander G.A., Franke L., Magalhães J.P. (2018). Gene co-expression analysis for functional classification and gene-disease predictions. Brief. Bioinform..

[7] Marbach D., Lamparter D., Quon G., Kellis M., Kutalik Z., Bergmann S. (2016). Tissue-specific regulatory circuits reveal variable modular perturbations across complex diseases. Nat. Methods.

[8] Albert R., Jeong H., Barabási A.L. (2000). Error and attack tolerance of complex networks. Nature.

[9] Luo M., Jiao J., Wang R. (2019). Screening drug target combinations in disease-related molecular networks. BMC Bioinform..

[10] Muhammad J., Khan A., Ali A., Fang L., Yanjing W., Xu Q., Wei D.Q. (2018). Network Pharmacology: Exploring the Resources and Methodologies. Curr. Top. Med. Chem..

[11] Le D.H. (2021). A network-based method for predicting disease-associated enhancers. PLoS ONE.

[12] Goh K.I., Cusick M.E., Valle D., Childs B., Vidal M., Barabási A.L. (2007). The human disease network. Proc. Natl. Acad. Sci. USA.

[13] Yan T., Ding F., Zhao Y. (2019). Integrated identification of key genes and pathways in Alzheimer’s disease via comprehensive bioinformatical analyses. Hereditas.

[14] Tong Z., Zhou Y., Wang J. (2019). Identifying potential drug targets in hepatocellular carcinoma based on network analysis and one-class support vector machine. Sci. Rep..

[15] Rodriguez-Esteban R., Jiang X. (2017). Differential gene expression in disease: A comparison between high-throughput studies and the literature. BMC Med. Genom..

[16] Le D.H. (2020). Machine learning-based approaches for disease gene prediction. Brief. Funct. Genom..

[17] Li X., Li W., Zeng M., Zheng R., Li M. (2020). Network-based methods for predicting essential genes or proteins: A survey. Brief. Bioinform..

[18] Liu J., Hua P., Hui L., Zhang L.L., Hu Z., Zhu Y.W. (2016). Identification of hub genes and pathways associated with hepatocellular carcinoma based on network strategy. Exp. Ther. Med..

[19] Bánky D., Iván G., Grolmusz V. (2013). Equal opportunity for low-degree network nodes: A PageRank-based method for protein target identification in metabolic graphs. PLoS ONE.

[20] Lei X., Wang S., Wu F. (2019). Identification of essential proteins based on improved HITS algorithm. Genes.

[21] Foutch D., Pham B., Shen T. (2021). Protein conformational switch discerned via network centrality properties. Comput. Struct. Biotechnol. J..

[22] Gosak M., Markovič R., Dolenšek J., Slak R.M., Marhl M., Stožer A., Perc M. (2018). Network science of biological systems at different scales: A review. Phys. Life Rev..

[23] Lv Y., Huang S., Zhang T., Gao B. (2021). Application of Multilayer Network Models in Bioinformatics. Front. Genet..

[24] Zhou G., Li S., Xia J. (2020). Network-based approaches for multi-omics integration. Comput. Methods Data Anal. Metab..

[25] Cantini L., Medico E., Fortunato S., Caselle M. (2015). Detection of gene communities in multi-networks reveals cancer drivers. Sci. Rep..

[26] Pournoor E., Mousavian Z., Dalini A.N., Masoudi N.A. (2020). Identification of key components in colon adenocarcinoma using transcriptome to interactome multi-layer framework. Sci. Rep..

[27] Mahapatra S., Bhuyan R., Das J., Swarnkar T. (2021). Integrated multiplex network based approach for hub gene identification in oral cancer. Heliyon.

[28] Zhang Y., Chen J., Wang Y., Wang D., Cong W., Lai B.S., Zhao Y. (2019). Multi-layer network analysis of miRNA and protein expression profiles in breast cancer patients. PloS ONE.

[29] Wang D., Wang H., Zou X. (2017). Identifying key nodes in multi-layer networks based on tensor decomposition. Chaos.

[30] Chen X., Xu M., An Y. (2021). Identifying the essential nodes in network pharmacology based on multilayer network combined with random walk algorithm. J. Biomed. Inform..

[31] Sanchez V.F., Mina M., Armenia J., Chatila W.K., Luna A., La K.C., Dimitriadoy S., Liu D.L., Kantheti H.S., Saghafinia S. (2018). Oncogenic Signaling Pathways in The Cancer Genome Atlas. Cell.

[32] The Cancer Genome Atlas Research Network (2014). Comprehensive molecular characterization of urothelial bladder carcinoma. Nature.

[33] Lin Y., Liu T., Cui T., Wang Z., Zhang Y., Tan P., Huang Y., Yu J., Wang D. (2020). RNAInter in 2020: RNA interactome repository with increased coverage and annotation. Nucleic Acids Res..

[34] Wei G., Dong Y., He Z., Qiu H., Wu Y., Chen Y. (2021). Identification of hub genes and construction of an mRNA-miRNA-lncRNA network of gastric carcinoma using integrated bioinformatics analysis. PLoS ONE.

[35] Hu W.L., Zhou X.H. (2017). Identification of prognostic signature in cancer based on DNA methylation interaction network. BMC Med. Genom..

[36] Wang J., Yang J., Li D., Li J. (2021). Technologies for targeting DNA methylation modifications: Basic mechanism and potential application in cancer. Biochim. Biophys. Acta BBA Rev. Cancer.

[37] Győrffy B., Bottai G., Fleischer T., Munkácsy G., Budczies J., Paladini L., Børresen-Dale A.L., Kristensen V.N., Santarpia L. (2016). Aberrant DNA methylation impacts gene expression and prognosis in breast cancer subtypes. Int. J. Cancer.

[38] Liang Y., Zhang C., Dai D.Q. (2019). Identification of differentially expressed genes regulated by methylation in colon cancer based on bioinformatics analysis. World J. Gastroenterol..

[39] Ritchie M.E., Phipson B., Wu D.I., Hu Y., Law C.W., Shi W., Smyth G.K. (2015). limma powers differential expression analyzes for RNA-sequencing and microarray studies. Nucleic Acids Res..

[40] Cui Z.J., Zhou X.H., Zhang H.Y. (2019). DNA methylation module network-based prognosis and molecular typing of cancer. Genes.

[41] Benedetti E., Pučić-Baković M., Keser T., Gerstner N., Büyüközkan M., Štambuk T., Selman M.H., Rudan I., Polašek O., Hayward C. (2020). A strategy to incorporate prior knowledge into correlation network cutoff selection. Nat. Commun..

[42] Kim K.S., Jekarl D.W., Yoo J., Lee S., Kim M., Kim Y. (2021). Immune gene expression networks in sepsis: A network biology approach. PLoS ONE.

[43] Zhang X., Wu D., Chen L., Li X., Yang J., Fan D., Dong T., Liu M., Tan P., Xu J. (2014). RAID: A comprehensive resource for human RNA-associated (RNA–RNA/RNA–protein) interaction. RNA.

[44] Guttman M., Rinn J.L. (2012). Modular regulatory principles of large non-coding RNAs. Nature.

[45] Wu X., Yang L., Wang J., Hao Y., Wang C., Lu Z. (2022). The Involvement of Long Non-Coding RNAs in Glioma: From Early Detection to Immunotherapy. Front. Immunol..

[46] Freeman L.C. (1978). Centrality in social networks conceptual clarification. Soc. Netw..

[47] Freeman L.C. (1977). A set of measures of centrality based on betweenness. Sociometry.

[48] Grobelny B.T., London D., Hill T.C., North E., Dugan P., Doyle W.K. (2018). Betweenness centrality of intracranial electroencephalography networks and surgical epilepsy outcome. Clin. Neurophysiol..

[49] Newman M.E. (2001). The structure of scientific collaboration networks. Proc. Natl. Acad. Sci. USA.

[50] Hao D., Ren C., Li C. (2012). Revisiting the variation of clustering coefficient of biological networks suggests new modular structure. BMC Syst. Biol..

[51] Ren Y., Ay A., Kahveci T. (2018). Shortest path counting in probabilistic biological networks. BMC Bioinform..

[52] Wallis K.F. (2003). Chi-squared tests of interval and density forecasts, and the Bank of England’s fan charts. Int. J. Forecast..

[53] Du S., Guo Y., Huang J., Xu J., Chen G. (2022). The Expressions and Functions of lncRNA Related to m6A in Hepatocellular Carcinoma from a Bioinformatics Analysis. Comput. Math. Methods Med..

[54] Bland J.M., Altman D.G. (2004). The logrank test. BMJ.

[55] Su P., Wen S., Zhang Y., Li Y., Xu Y., Zhu Y., Lv H., Zhang F., Wang M., Tian Z. (2016). Identification of the Key Genes and Pathways in Esophageal Carcinoma. Gastroenterol. Res. Pract..

[56] Smith J.J., Deane N.G., Wu F., Merchant N.B., Zhang B., Jiang A., Lu P., Johnson J.C., Schmidt C., Bailey C.E. (2010). Experimentally derived metastasis gene expression profile predicts recurrence and death in patients with colon cancer. Gastroenterology.

[57] Alvord W.G., Roayaei J., Stephens R. (2007). The DAVID gene functional classification tool: A novel biological module-centric algorithm to functionally analyze large gene lists. Genome Biol..

[58] Chang H.C., Chu C.P., Lin S.J., Hsiao C.K. (2020). Network hub-node prioritization of gene regulation with intra-network association. BMC Bioinform..

[59] Ashtiani M., Salehzadeh Y.A., Razaghi M.Z., Hennig H., Wolkenhauer O., Mirzaie M., Jafari M. (2018). A systematic survey of centrality measures for protein-protein interaction networks. BMC Syst. Biol..

[60] Duffy M.J., O’Grady S., Tang M., Crown J. (2021). MYC as a target for cancer treatment. Cancer Treat. Rev..

[61] Ijaz M., Wang F., Shahbaz M., Jiang W., Fathy A.H., Nesa E.U. (2018). The Role of Grb2 in Cancer and Peptides as Grb2 Antagonists. Protein Pept. Lett..

[62] Gao Y., Feng B., Lu L., Han S., Chu X., Chen L., Wang R. (2017). MiRNAs and E2F3: A complex network of reciprocal regulations in human cancers. Oncotarget.

[63] Nebenfuehr S., Kollmann K., Sexl V. (2020). The role of CDK6 in cancer. Int. J. Cancer.

[64] Poluri R.T., Paquette V., Allain É.P., Lafront C., Joly B.C., Weidmann C., Droit A., Guillemette C., Pelletier M., Audet W.É. (2021). KLF5 and NFYA factors as novel regulators of prostate cancer cell metabolism. Endocr. Relat. Cancer.

[65] Burgess J.T., Bolderson E., Adams M.N., Duijf P.H.G., Zhang S.D., Gray S.G., Wright G., Richard D.J., O’Byrne K.J. (2020). SASH1 is a prognostic indicator and potential therapeutic target in non-small cell lung cancer. Sci. Rep..

[66] Xiong H., Chen Z., Zheng W., Sun J., Fu Q., Teng R., Chen J., Xie S., Wang L., Yu X.F. (2020). FKBP4 is a malignant indicator in luminal A subtype of breast cancer. J. Cancer.

[67] Verma M., Patel P., Verma M. (2011). Biomarkers in prostate cancer epidemiology. Cancers.

[68] Chu Y., Lai Y.H., Lee M.C., Yeh Y.J., Wu Y.K., Tsao W., Huang C.Y., Wu S. (2017). Calsyntenin-1, clusterin and neutrophil gelatinase-associated lipocalin are candidate serological biomarkers for lung adenocarcinoma. Oncotarget.

[69] Humbert P.O., Pryjda T.Z., Pranjic B., Farrell A., Fujikura K., Dematos S.R., Karim R., Kozieradzki I., Cronin S.J.F., Neely G.G. (2022). TSPAN6 is a suppressor of Ras-driven cancer. Oncogene.

[70] Sun S., Dammann J., Lai P., Tian C. (2022). Thorough statistical analyses of breast cancer co-methylation patterns. BMC Genom. Data.

[71] Kundu A., Nam H., Shelar S., Chandrashekar D.S., Brinkley G., Karki S., Mitchell T., Livi C.B., Buckhaults P., Kirkman R. (2020). PRDM16 suppresses HIF-targeted gene expression in kidney cancer. J. Exp. Med..

[72] Sengelaub C.A., Navrazhina K., Ross J.B., Halberg N., Tavazoie S.F. (2016). PTPRN 2 and PLC\beta1 promote metastatic breast cancer cell migration through PI (4, 5) P2-dependent actin remodeling. EMBO J..

[73] Yin J., Guo Y. (2021). HOXD13 promotes the malignant progression of colon cancer by upregulating PTPRN2. Cancer Med..

[74] Eckhardt F., Lewin J., Cortese R., Rakyan V.K., Attwood J., Burger M., Burton J., Cox T.V., Davies R., Down T.A. (2006). DNA methylation profiling of human chromosomes 6, 20 and 22. Nat. Genet..

[75] Huang J. (2021). Current developments of targeting the p53 signaling pathway for cancer treatment. Pharmacol. Ther..

[76] Espinoza N.A., Goette M. (2020). Role of cell surface proteoglycans in cancer immunotherapy. Semin. Cancer Biol..

[77] Ediriweera M.K., Tennekoon K.H., Samarakoon S.R. (2019). Role of the PI3K/AKT/mTOR signaling pathway in ovarian cancer: Biological and therapeutic significance. Semin. Cancer Biol..

