# Supplementary figures and images for: Identifying Tumor-Associated Genes from Bilayer Networks of DNA Methylation Sites and RNAs
Source: Life (Basel). 2022 Dec 27;13(1):76. doi: 10.3390/life13010076 (PMC9861397; doi:10.3390/life13010076)

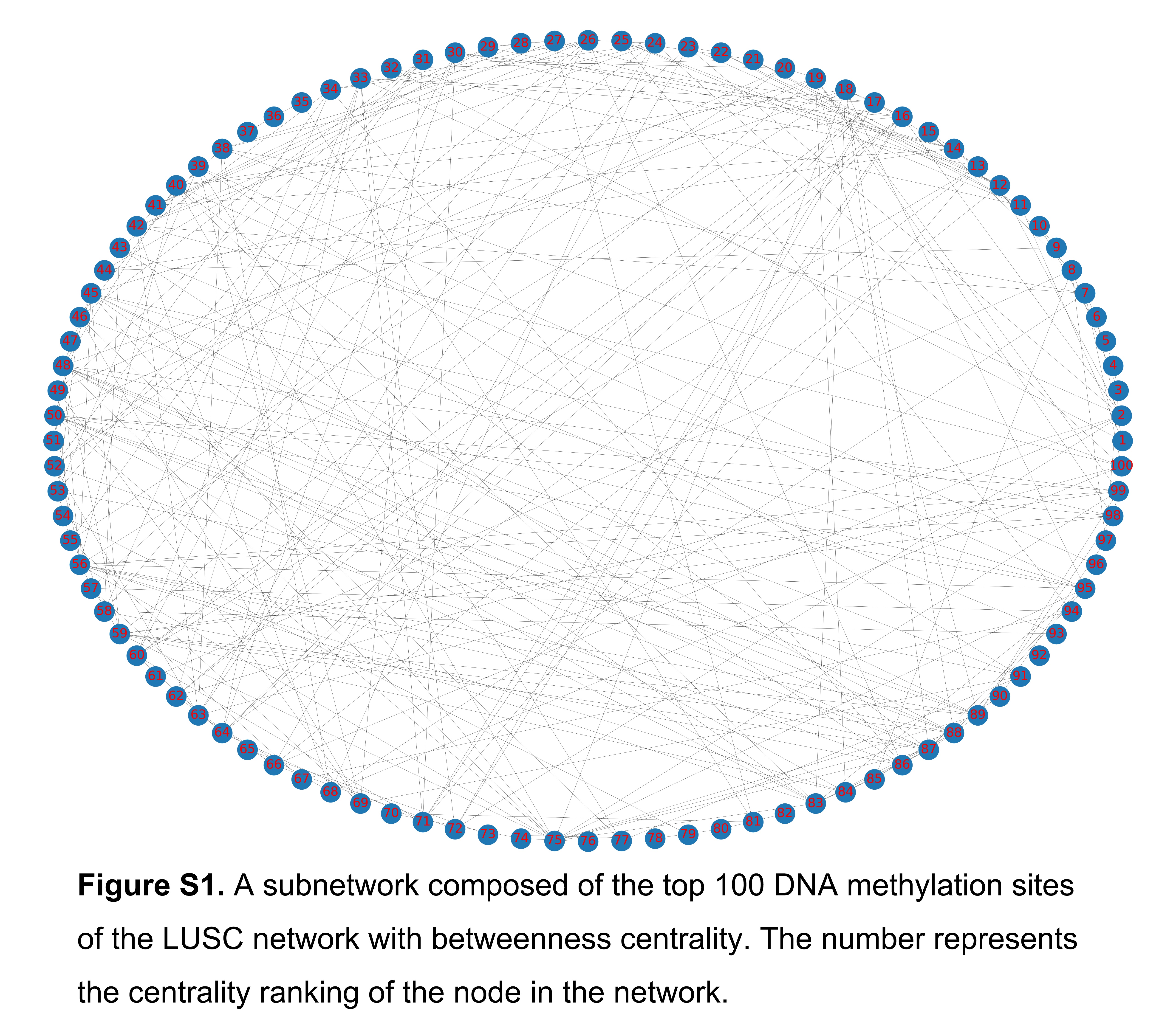

Supplement: Supplementary file 1 [file life-13-00076-s001.zip › life-1989777-supplementary/Figure S1. Hub nodes found by the betweenness centrality constitute the subnetwork of the LUSC.jpg]

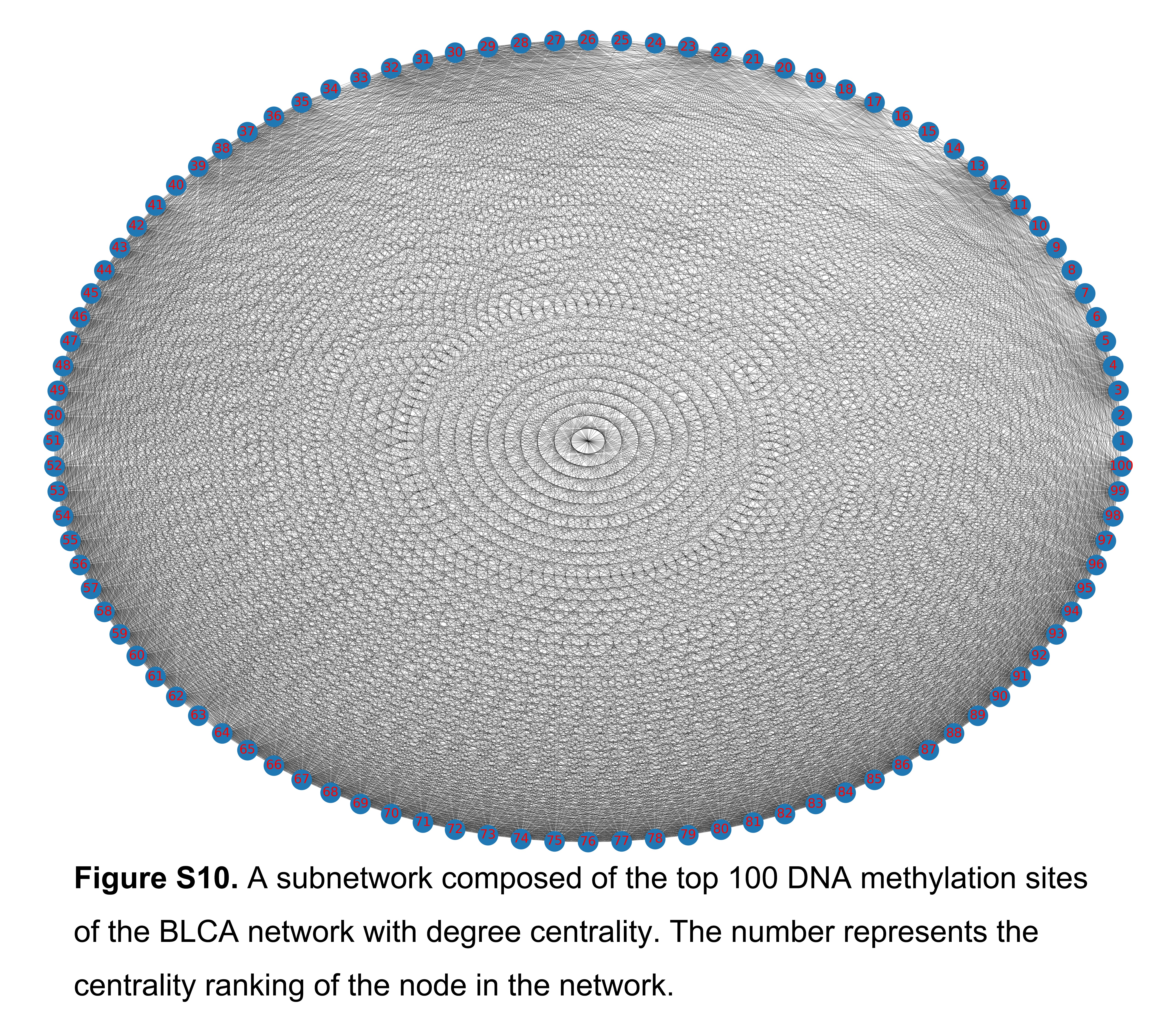

Supplement: Supplementary file 1 [file life-13-00076-s001.zip › life-1989777-supplementary/Figure S10. Hub nodes found by the degree centrality constitute the subnetwork of the BLCA.jpg]

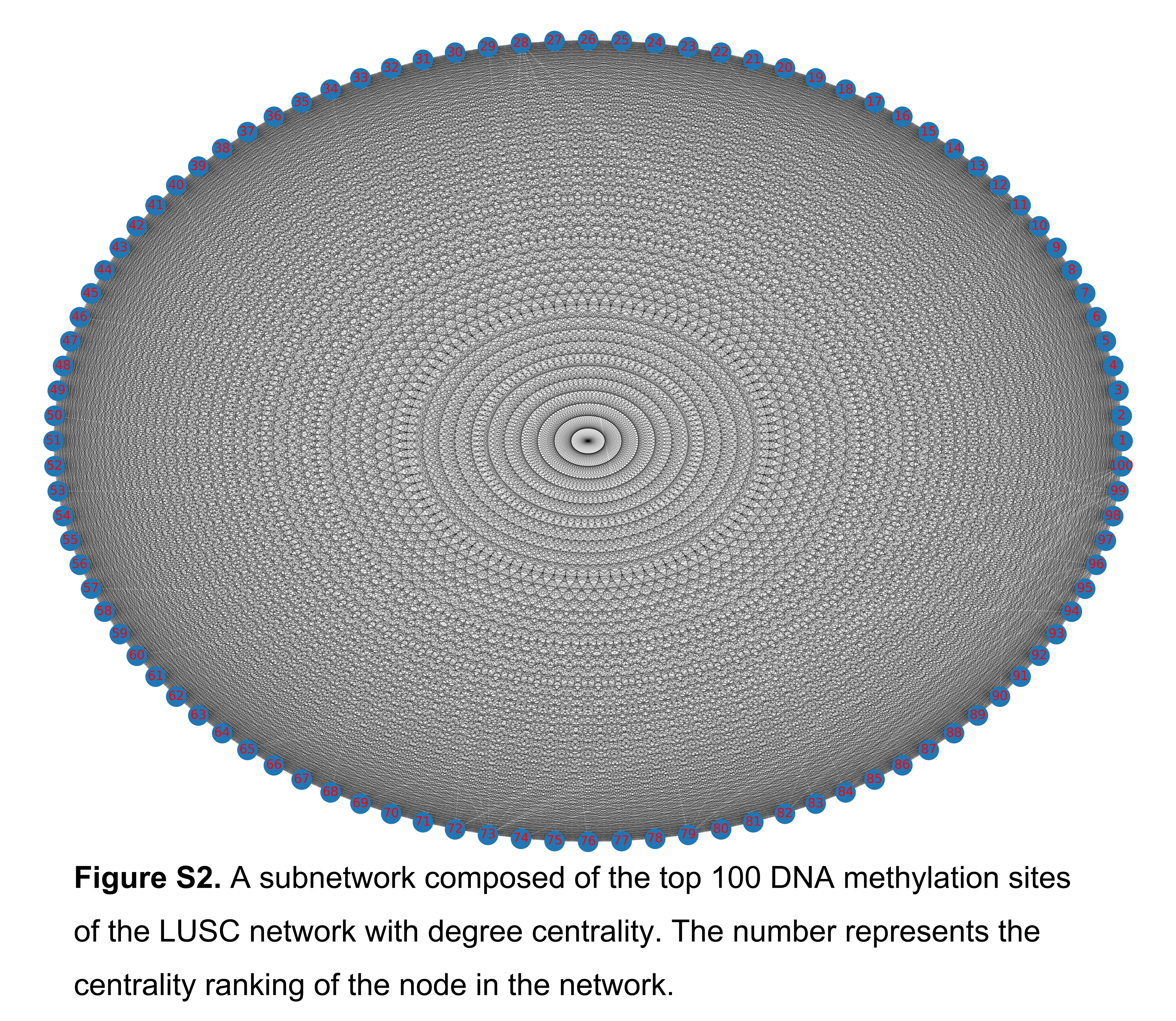

Supplement: Supplementary file 1 [file life-13-00076-s001.zip › life-1989777-supplementary/Figure S2. Hub nodes found by the degree centrality constitute the subnetwork of the LUSC.jpg]

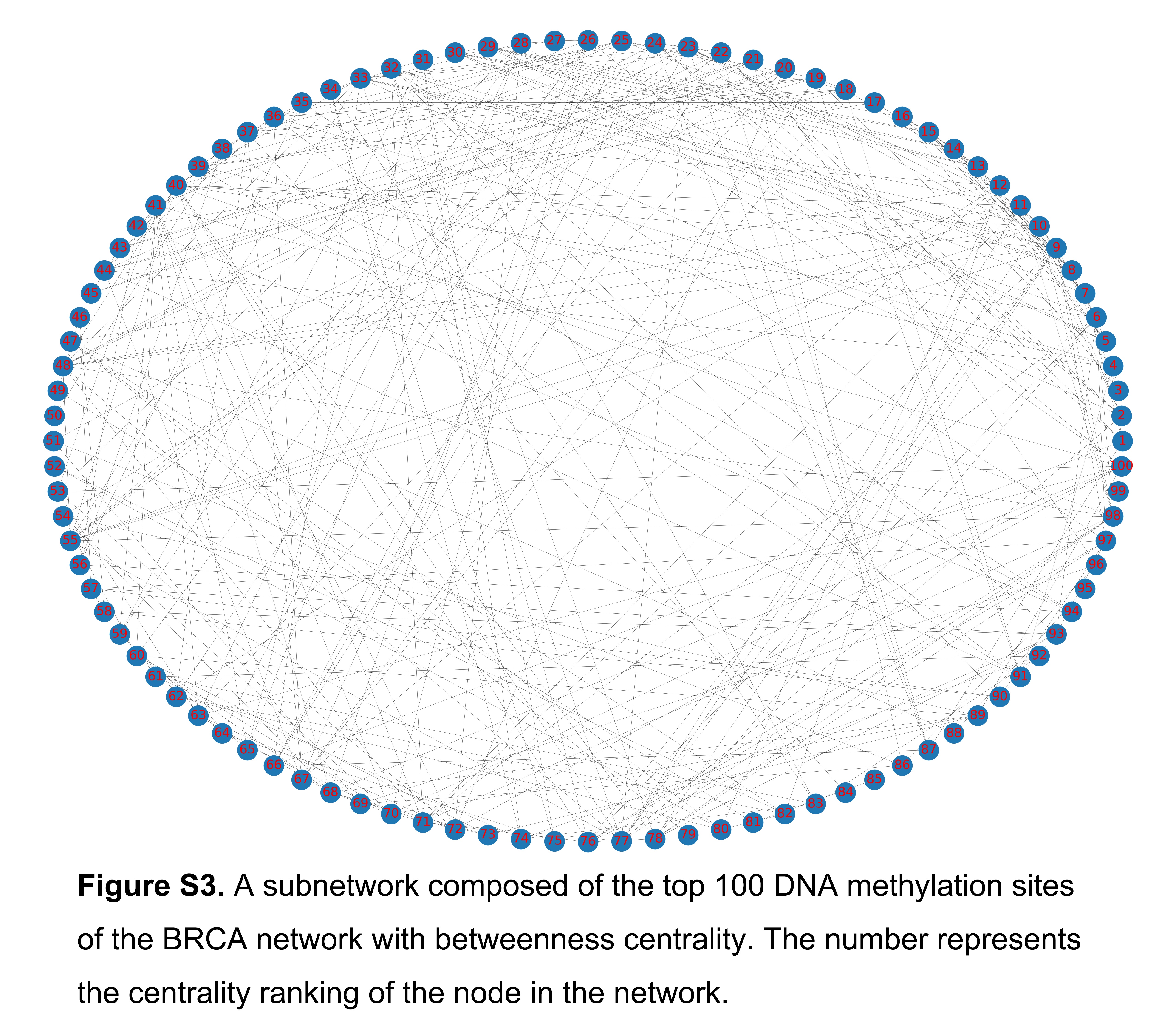

Supplement: Supplementary file 1 [file life-13-00076-s001.zip › life-1989777-supplementary/Figure S3. Hub nodes found by the betweenness centrality constitute the subnetwork of the BRCA.jpg]

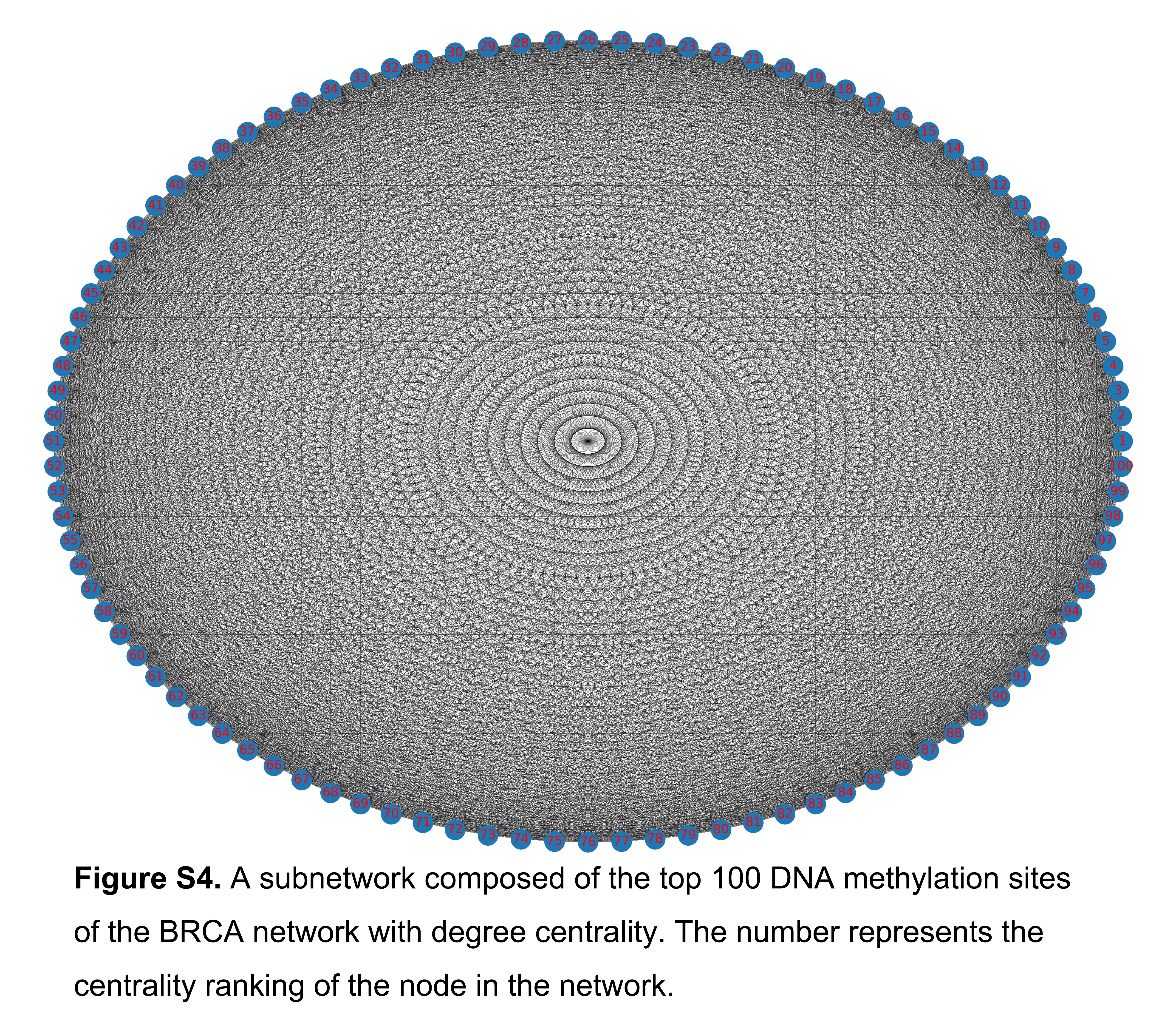

Supplement: Supplementary file 1 [file life-13-00076-s001.zip › life-1989777-supplementary/Figure S4. Hub nodes found by the degree centrality constitute the subnetwork of the BRCA.jpg]

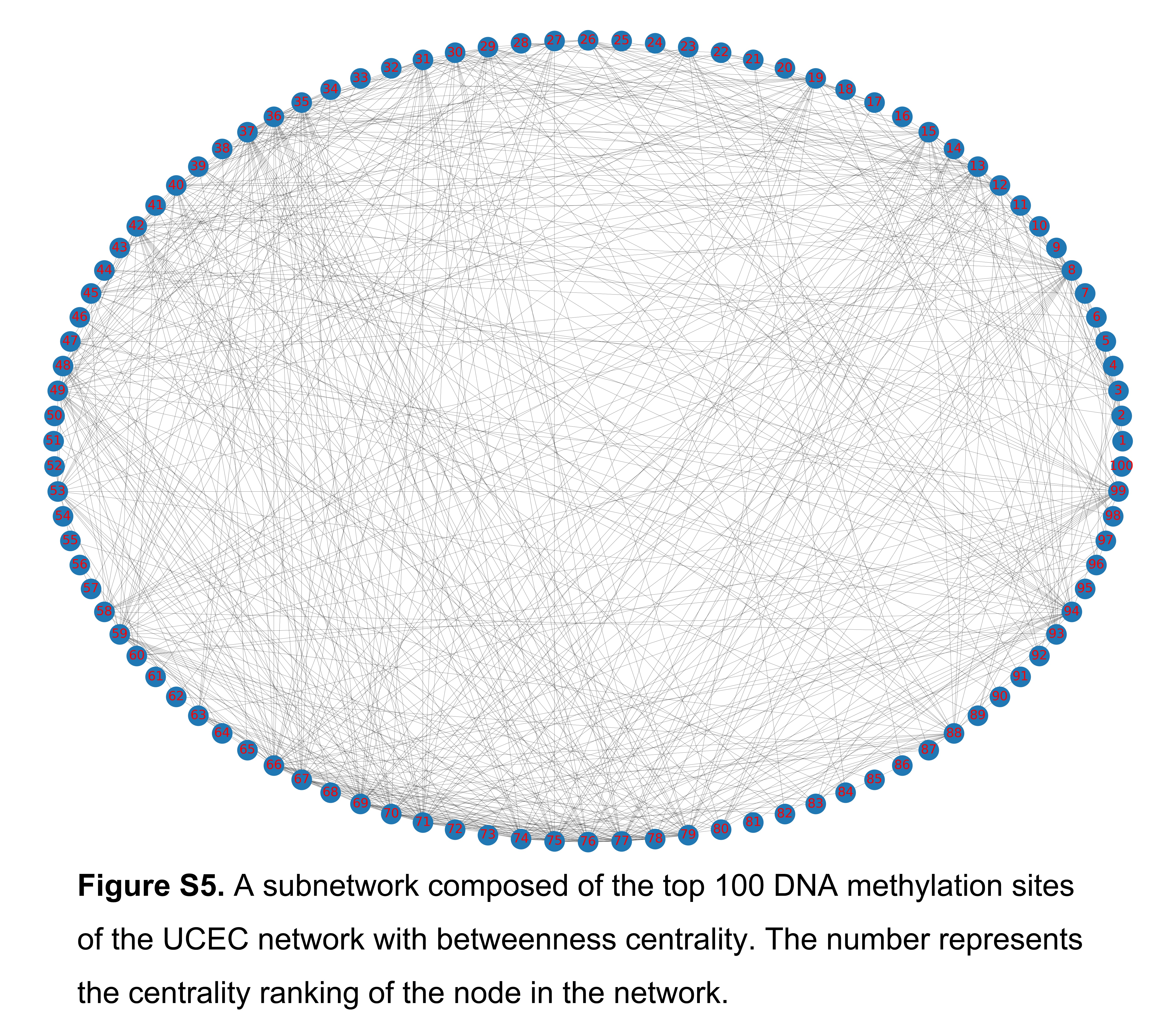

Supplement: Supplementary file 1 [file life-13-00076-s001.zip › life-1989777-supplementary/Figure S5. Hub nodes found by the betweenness centrality constitute the subnetwork of the UCEC.jpg]

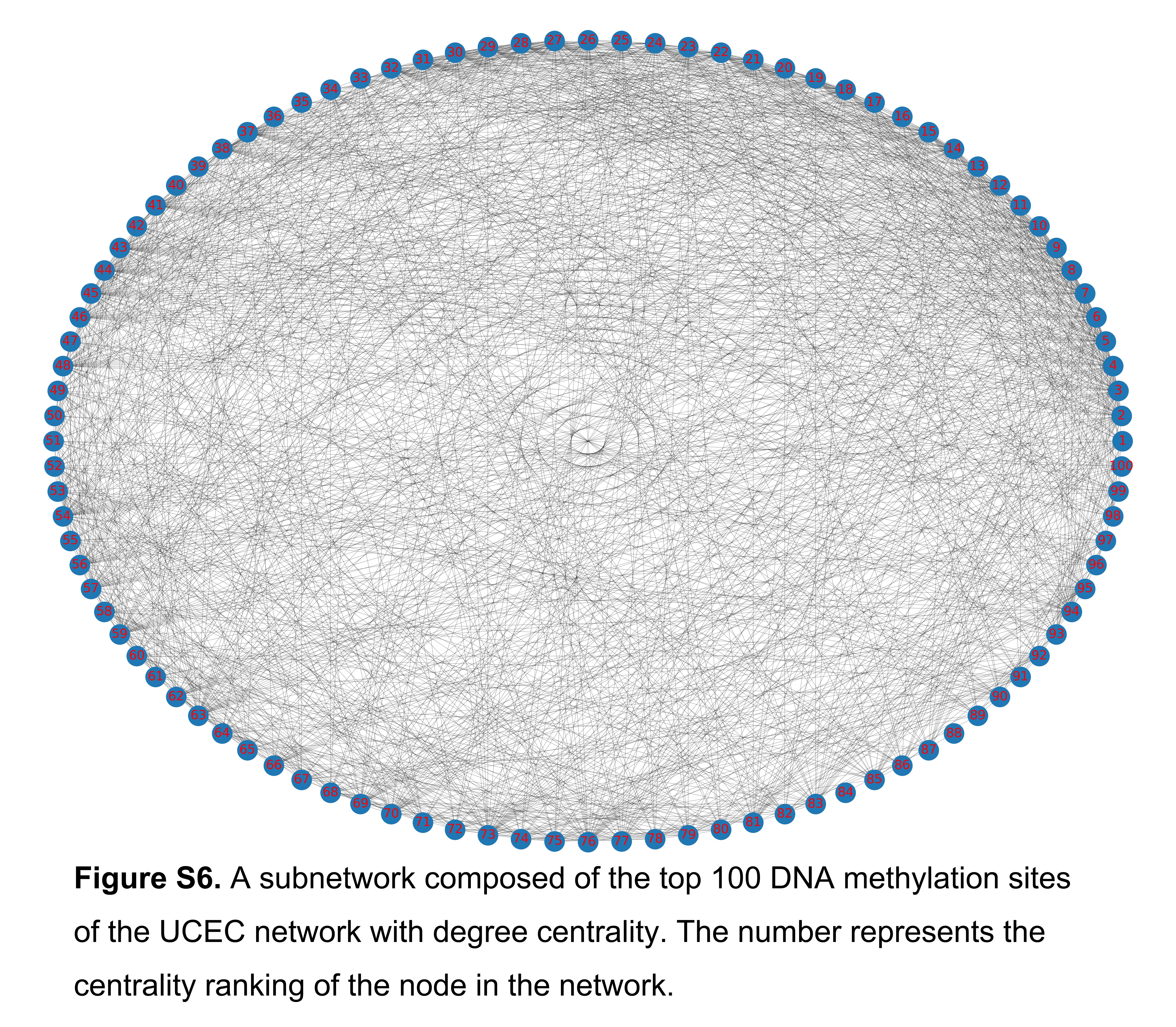

Supplement: Supplementary file 1 [file life-13-00076-s001.zip › life-1989777-supplementary/Figure S6. Hub nodes found by the degree centrality constitute the subnetwork of the UCEC.jpg]

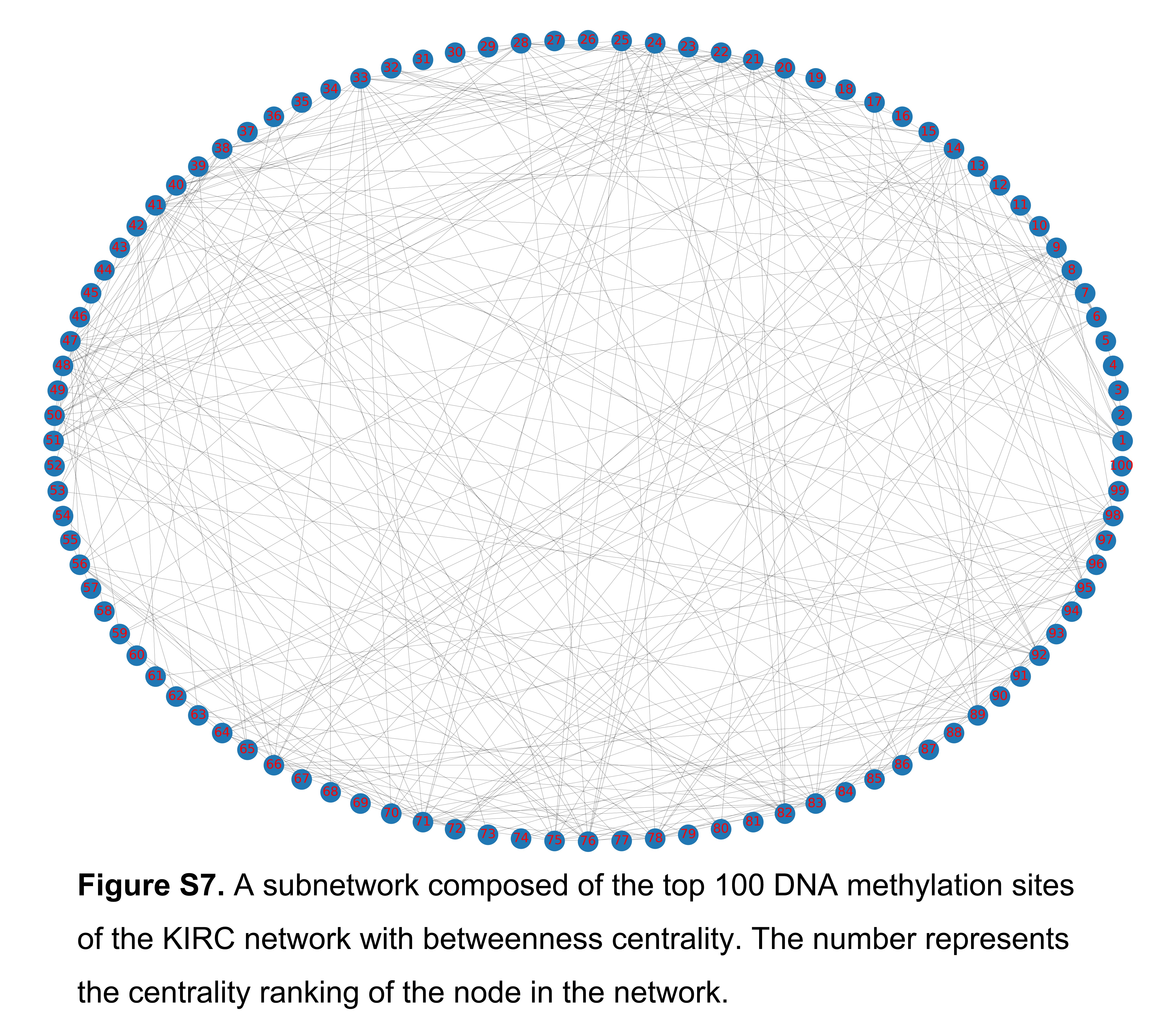

Supplement: Supplementary file 1 [file life-13-00076-s001.zip › life-1989777-supplementary/Figure S7. Hub nodes found by the betweenness centrality constitute the subnetwork of the KIRC.jpg]

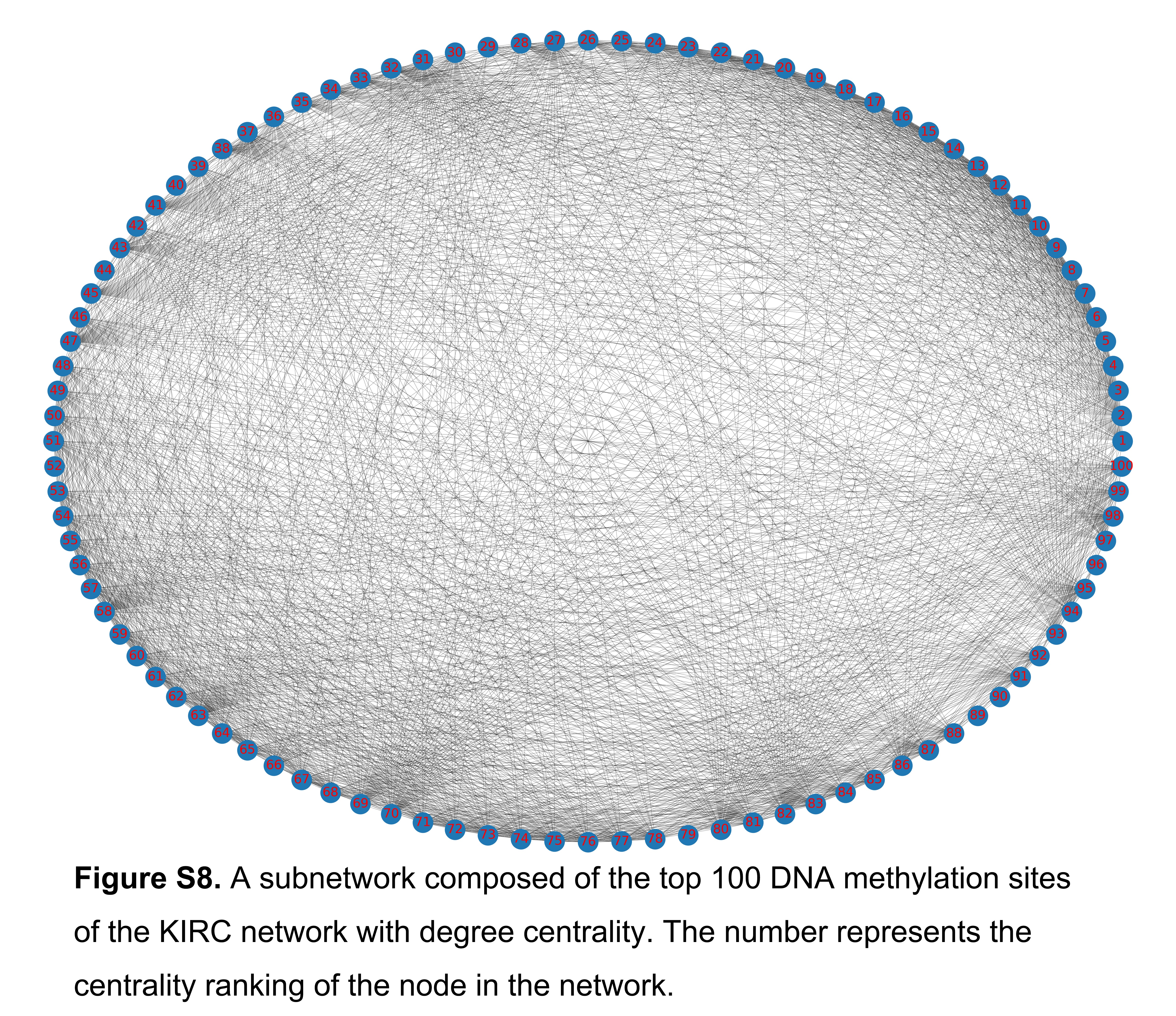

Supplement: Supplementary file 1 [file life-13-00076-s001.zip › life-1989777-supplementary/Figure S8. Hub nodes found by the degree centrality constitute the subnetwork of the KIRC.jpg]

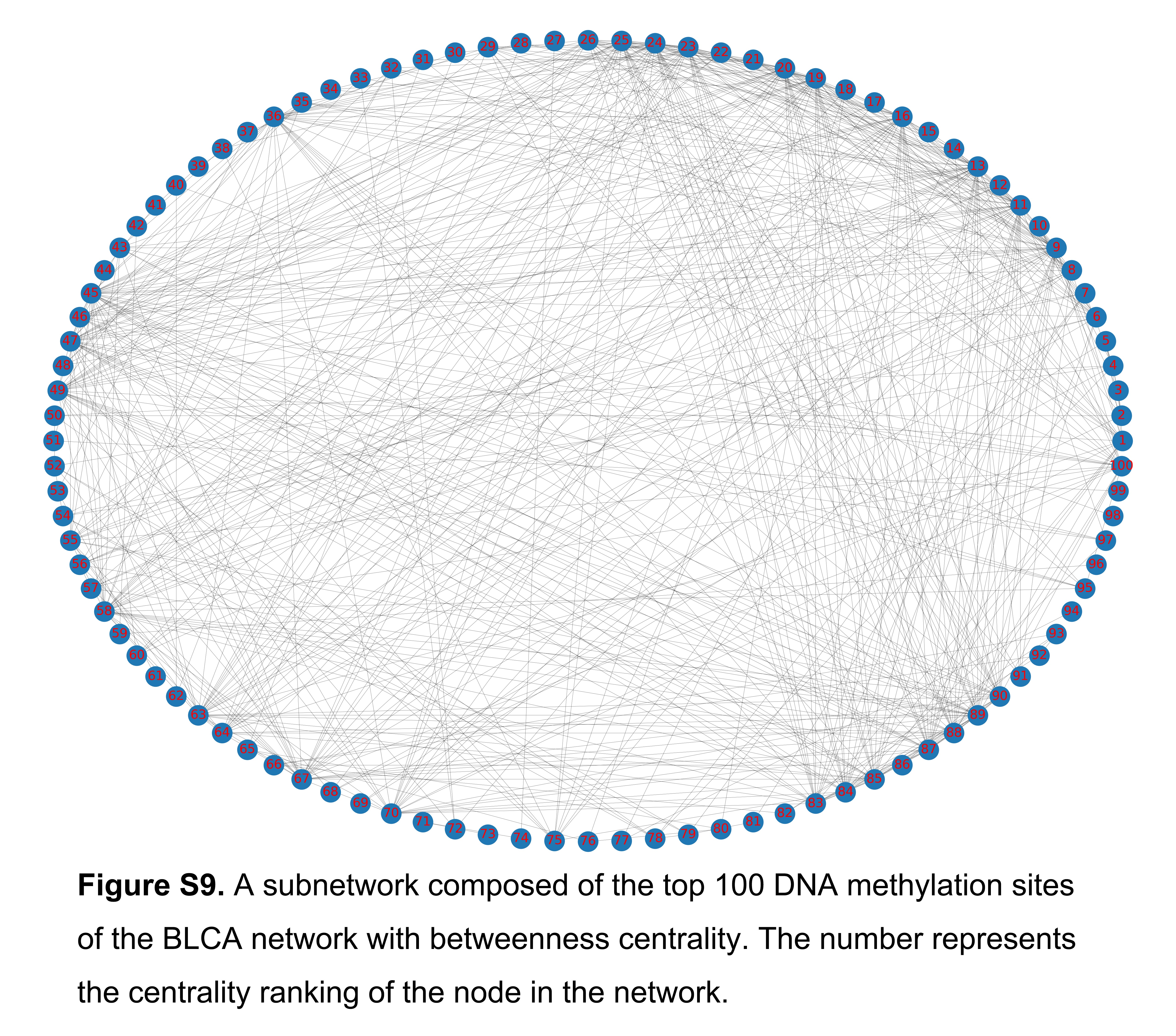

Supplement: Supplementary file 1 [file life-13-00076-s001.zip › life-1989777-supplementary/Figure S9. Hub nodes found by the betweenness centrality constitute the subnetwork of the BLCA.jpg]
